# Evaluation of potential of Zn-pectinate gel (ZPG) microparticles containing mesalazine for colonic drug delivery

**Published:** 2010

**Authors:** J. Kawadkar, K. Chauhan Meenakshi, A. Ram

**Affiliations:** 1 Delhi Institute of Pharmaceutical Sciences and Research (DIPSAR), University of Delhi, New Delhi; 2 SLT Institute of Pharmaceutical Sciences, Guru Ghasidas Central University, Bilaspur, India

**Keywords:** Pectin Microparticles (MPs), Mesalazine, Colonic drug delivery

## Abstract

**Background and the purpose of the study:**

Pectin derivatives have been utilized for colonic drug delivery (CDD). In this study the effects of different formulation variables upon the characteristics of pectinate microparticles (MPs) prepared by ionotropic gelation technique for colonic delivery of mesalazine was investigated.

**Methods:**

*In-vitro* drug release of MPs was studied using USP XXIV dissolution apparatus type I, in different fluids e.g. simulated gastric fluid (SGF: pH 1.2), simulated intestinal fluid (SIF: pH 7.4), and simulated colonic fluid (SCF: pH 6.8) of volume 900 ml, at 100 rpm maintained at 37±0.2°C. This study was also performed in the presence of 4% w/v rat caecal content (RCC) using phosphate buffer saline (pH 6.8) as SCF. Gamma scintigraphy study was performed on New Zealand rabbit animal model using ^99m^ Tc.

**Results:**

The results showed that maximum entrapment of mesalazine (86.1±1.7%) and strength of gel network zinc pectinate gel microparticles (ZPGD2) was achieved in cross-linking solution of pH 1.6. Batch of ZPGD2 showed least swelling ratio and drug release. In RCC medium the t_50%_ value of CPG-MPs was 3–4 folds greater than ZPG-MPs. Scintigram showed the residence of ZPG-MPs (filled in enteric coated capsule) in colon more than 9 hrs and delivery of almost all the drug loading dose in colon.

**Major conclusion:**

The results of this study suggest the designed formulation of ZPG-MPs has the potential to serve as a colonic drug delivery system.

## INTRODUCTION

Colonic drug delivery (CDD) is intended for the local treatment of ulcerative colitis, inflammatory bowel diseases and can potentially be used for colon cancer or the systemic administration of drugs that are adversely affected by the upper gastro-intestinal (GI) tract (1). CDD have advantages of minimum systemic absorption, administration of lower drug doses, high concentrations of drug in the distal small intestine and the colon without systemic exposure and toxicity. There has been considerable investigation for the design of CDD systems and targeting has been achieved by different methods. Delivery systems controlling release of drug only in the colon has been reported (2).

Mesalazine (MZ), an anti-inflammatory drug, prevents development of colorectal cancer and delivery system is required. The rationale for the development of a polysaccharide based delivery system for colon is the ability of the colonic microfolra to degrade various types of polysaccharides that escape small bowel digestion (6). Pectins are naturally occurring biodegradable polysaccharides consisting of linear chain of 1–4 linked α-D-galacturonic acid residues with varying degrees of methyl ester substituents (7). They are broken down by various microbial sources including human colonic bacteria and may therefore be utilized as CDD systems if their solubility are reduced. Major efforts have been focused should be deleted for pectin derivatives (8) and use of calcium and zinc salts of pectin because binding of calcium and zinc induces non-covalent associations of carbohydrate chains through “egg- box” complexes (9) which are more water resistant, ulcerative colitis (3, 4). MZ (t1/2: 0.6 to 1.4 hrs) is while still enzymatically degradable. In this study rapidly absorbed from the small intestine and there is a little localization in the colon relative to the small intestine (5). Therefore, to increase its localization in the colon; development of a polysaccharide based the effects of different formulation variables upon the characteristics of pectinate microparticles (MPs) prepared by ionotropic gelation technique for colonic delivery of mesalazine was investigated.

## MATERIAL AND METHODS

### 

#### Materials

Mesalazine, Pure pectin, and SnCl_2_ were purchased from HiMedia Laboratory Pvt. Ltd, Mumbai (India). Amidated low methoxy (LM) pectin (29.7% degree of esterification and 13.3% degree of amidation) were prepared in author's laboratory by the reported method (10). Zn(CH_3_ COO)_2_ and CaCl_2_ dihydrate were purchased from Central Drug House Pvt. Ltd, New Delhi (India). ^99m^ Tc as pertechnetate (^99m^TcO−_4_) for radiolabeling of MPs was obtained from the Nuclear Medicine Department, Jawaharlal Nehru Cancer Hospital and Research Center, Bhopal (India). All should be deleted other chemicals and solvents were of analytical reagent grade.

The in vivo study was performed by a protocol approved by the Committee for the Purpose of Control and Supervision of Experiments on Animals, Ministry of Social Justice and Empowerment, Government of India. Animal Ethical committee of Jawaharlal Nehru Cancer Hospital and Research Centre, Bhopal (India) granted permission for the Hospital and Research Centre, Bhopal (India).

#### Preparation of MPs

The ionotropic gelation technique, (11–13) was used with slight modifications. Aqueous dispersion of LM-pectin (2% or 4% w/v) was prepared by overnight agitation. A specified amount of MZ (1% w/v) was dispersed into the agitated polymer dispersion until a uniform dispersion was obtained. This homogenous, and bubble-free dispersion was dropped through a disposable syringe (0.6 mm inner diameter of nozzle) at an average rate of 1 ml/min, into 50 ml solution of cross-linking agent (0.1 M of Zn(CH_3_COO)_2_ or 0.15 M of CaCl_2_ dihydrate) agitated at 150 rpm. The falling distance was 5 cm. The pH of this solution was adjusted at 1.6, 3.8 and 6.1 by 1 N HCl. The formed MPs gelles were allowed to stand in the cross-linking solution for 6 to 24 hrs. The MPs were separated by filtration, washed three times with deionized water and dried at 37 °C for 48 hrs in a drying room. Different variables were investigated and are summarised in table 1.

**Table 1:** Various formulations of microparticles.

Formulation code	LM-Pectin Conc. (% w/v)	Zn(CH_3_COO)_2_ (0.1 M) pH	CaCl_2_ (0.15 M) pH	Cross-linking time (hrs)
ZPGA1	2	1.6	—	6
ZPGA2	4	1.6	—	6
ZPGB1	2	3.8	—	6
ZPGB2	4	3.8	—	6
ZPGC1	2	6.1	—	6
ZPGC2	4	6.1	—	6
ZPGD1	4	1.6	—	12
ZPGD2	4	1.6	—	24
CPG	4	—	1.6	24

*ZPG and CPG indicate zinc pectinate gel microparticles and calcium pectinate gel icroparticles respectively

#### Determination of size distribution of MPs

Particle size and size distribution of different formulations of zinc pectinate gel microparticles (ZPG-MPs) and calcium pectinate gel microparticles (CPG-MPs) were measured using an optical microscope with calibrated ocular micrometer and the mean particle size was calculated by measurement of 300 particles of each formulation.

#### Entrapment efficiency (EE)

A weighed amount of MZ loaded MPs was suspended in phosphate buffer (pH 6.8) and magnetically stirred at 200 rpm for 24 hrs to promote swelling and breakup of the cross-linked structure. This solution was vacuum filtered through a 0.45 µm membrane filter (Millipore Corp, MA) and the absorbance of the resulting solution was measured at 330 nm using UV-spectrophotometer (Systronic, Japan) to determine the entrapment efficiency.

#### Swellability of MPs

MPs (100 mg) were placed in little excess of simulated colonic fluid (SCF: KH_2_ PO_4_ /NaOH buffer, pH 6.8) and allowed to swell for the requiredperiod of time using the dissolution apparatus with the dissolution basket assembly at 100 rpm and 37±0.2 °C. The MPs were periodically removed at pre-determined intervals, blotted with filter paper and their changes in weight (after correction for drug loss) were measured during the swelling until equilibrium was attained. Finally, the weight of the swollen MPs was recorded and the swelling ratio(SR) was then calculated from the formula:

SR=[(Wf-Wi)/Wi]

where Wi is the initial weight of the MPs and Wf is the corrected final weight of the MPs at equilibrium swelling in the medium.

#### In vitro drug release studies

The drug release studies of the MPs equivalent to 50 mg of MZ were performed using USP XXIV type I dissolution test apparatus (Electrolab, India) at 100 rpm and 37±0.2 °C in 900 ml simulated GI fluids. MPs were filled in hard gelatin capsules and coated with optimized eudragit S 100 (5%, four coatings) and then drug release were studied in simulated gastric fluid (SGF: HCl/NaCl buffer, pH 1.2) for the first 2 hrs. Then, the dissolution medium was replaced with simulated intestinal fluid (SIF: KH_2_PO_4_/NaOH buffer, pH 7.4) and tested for drug release for 3 hrs, and finally SCF (KH_2_PO4/NaOH buffer, pH 6.8) was used for 7 hrs to mimic colonic pH conditions. This pH status was found upon physiological data (6). Periodically samples were withdrawn and replaced with fresh buffer. The withdrawn samples were filtered and in the filtrate MZ content was measured by UV-spectrophotometer (Systronic, Japan) at 302 nm for pH 1.2 media, and 330 nm for pH 7.4 and 6.8 media respectively.

#### In vitro drug release study in the presence of rat caecal content (RCC)

The susceptibility of the pectinate MPs to the enzymatic action of colonic bacteria was assessed by continuing the drug release studies in RCC medium which was prepared by the reported method (6). Phosphate buffer saline (PBS) of pH 6.8, with 4% w/v RCC was used as SCF. The enteric coated capsules filled with 50 mg MZ loaded MPs were placed in 200 ml of this dissolution media (PBS, pH 6.8). The CO_2_ was supplied into the dissolution medium during the experiment. At different time intervals, the samples were withdrawn and replaced with fresh PBS. The experiment was continued up to 24 hrs. The withdrawn samples were centrifuged, the supernatant was filtered and the filtrate was analyzed for MZ content at 330 nm using UV-spectrophotometer (Systronic, Japan).

#### Fourier transform infrared spectroscopy (FT-IR)

Drug polymer interactions were studied by FT-IR. The spectra of different samples were recorded using FT-IR spectrophotometer (Perkin Elmer, Japan). Samples were prepared in KBr disks. Each KBr disk was scanned over a wave number region of 500 to 4000 cm^−1^.

#### Morphology

The shape and surface morphology of ZPG-MPs and CPG-MPs were investigated using scanning electron microscopy (SEM). The samples were prepared by sprinkling the MPs on a double adhesive tape stuck to an aluminum stub. The stubs were then coated with gold using a gold sputter module. The coated samples were scanned and photomicrographs were taken by a scanning electron microscope (Jeol JSM-1600, Japan).

#### Radiolabeling of MPs

SnCl_2_ (3% w/v) loaded ZPG-MPs (200 mg) were placed in the test tube and soaked in 10 ml of normal saline for 20 min. A small amount of ^99m^Tc solution (40 mBq radioactivity) from a Technetium generator (column generator, Monrol, Mon-tek, ^99^Mo^99^Tc, Turkey) was added to test tube. The suspension was mixed and MPs were allowed to equilibrate. The supernatant was removed and the labeled MPs were recovered by filtration followed by washing with deionized water and then air dried. Dried MPs were filled in gelatin capsules (# 4), and capsules were coated with optimized coating of eudragit S100 polymer (5%, four coatings), and air dried.

#### Gamma scintigraphy

Six male New Zealand rabbits (3 kg) were used to monitor the in vivo transit behavior of ZPG-MPs. None of animals had symptoms or a past history of GI disease. To standardize the condition of GI motility, animals were fasted for 12 hrs prior to the experiment. One enteric coated capsule of the ZPG- MPs was orally administered to each animal by a feeding tube, followed by a sufficient volume of drinking water. The location of the formulation in GI tract was monitored every one hours by keeping the anterior part of subject in front of gamma camera. Specific GI tract sites were imaged by E-Cam Single Head gamma camera (Siemen's, Germany). The gamma images were recorded for a 9 hrs study period.

#### Statistical analysis

Experimental data have been represented as the mean±s.d. of different independent determinations. The data of drug released before entrance in colon by ZPG-MPs and CPG-MPs were analysed using unpaired *t*-test at the significance level of *P* <0.0001.

## RESULT AND DISCUSSION

### 

#### Preparation, morphology, size and size distribution

According to “Egg-box model” (14) when the MZ containing aqueous dispersion of LM-pectin was dropped into cross-linking solutions (Zn^2+^ or Ca^2+^ counter-ions), gelled spheres were produced instantaneously. In this process, intermolecular cross-links were formed between the negatively charged carboxyl groups of LM-pectin and the positively charged counter-ions. Various formulations which were prepared are shown in table 1. SEM photomicrographs showed that ZPG- MPs and CPG-MPs had a spherical morphology.

**Figure 1:**
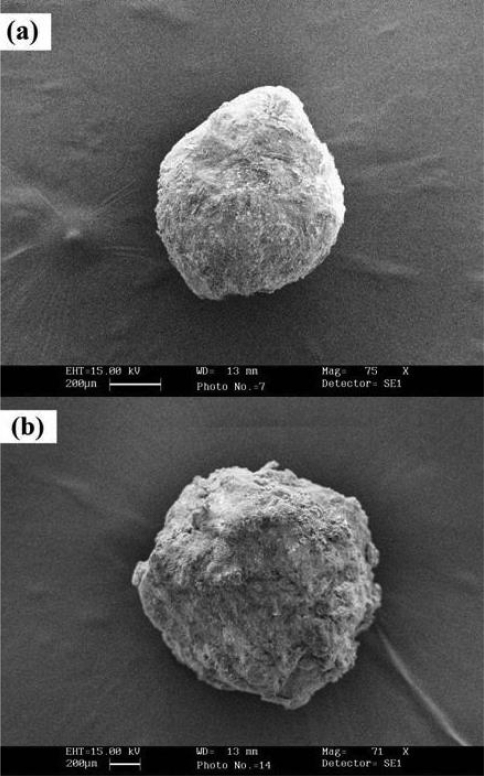
Scanning electron microscopy (SEM) of zinc pectinate gel microparticles (ZPG-MPs) (a), and calcium pectinate gel (microparticles (CPG-MPs) (b

However, the absence of ideal spherical morphology can be probably attributed to the drying process that causes certain invaginations in the particles. The surface of ZPG-MPs appeared globulous with low porosity. As for alginate beads, ZPG-MPs seemed to be smaller than CPG-MPs (15) (Figure 1(A) and (B)) because calcium ions form loose linkages with carboxyl groups in the chains of LM pectin and zinc ions form more extensive cross-linking due to its mutual interaction. Extensive cross-linking causes reduction in size and results are supported by the SEM images and optical microscopy. The prepared ZPG-MPs showed varied mean diameter from 526.07±6.38 µm to 688.93±4.39 µm. The mean diameter of CPG-MPs was found 723.51±4.53 µm. The average particle size of all formulations is shown in table 2.

**Table 2:** Particles size, entrapment efficiency (EE), % yield and swelling ratio in simulated colonic fluid (pH 6.8) of different formulation batches^a^.

Formulation Code	Particle size (µm)	EE (%)^b^	Yield (%)^c^	Swelling ratio at pH 6.8
ZPGA1	4.22±569.34	2.3±81.8	1.4±94.6	0.62±1.87
ZPGA2	7.87±583.18	1.2±82.1	1.3±96.7	0.60±2.68
ZPGB1	5.51±607.58	1.5±74.6	1.6±83.4	0.97±4.79
ZPGB2	6.31±624.63	1.9±75.1	1.9±85.7	1.24±5.22
ZPGC1	3.41±657.79	1.7±69.5	1.7±81.3	1.68±6.12
ZPGC2	4.39±688.93	2.3±71.2	2.6±81.9	1.79±6.83
ZPGD1	3.94±542.32	1.5±84.8	1.1±94.3	0.76±1.49
ZPGD2	6.38±526.07	1.7±86.1	2.1±97.6	0.37±0.88
CPG	4.53±723.51	1.3±83.7	2.5±92.3	1.73±9.17

a Results are mean±s.d. (n=3),

b EE (%)=Calculated drug content/Theoretical drug content × 100

c Yield (%)=Total weight of MPs/ total weight of drug and polymer × 100

#### Entrapment efficiency, percent yield and swelling ratio

EE,% yield and SR of different formulation batches are presented in table 2. The EE increased progressively by increase in polymer concentration when prepared at the pH of individual cross-linking solution but decreased by increase in pH of the cross-linking solution from 1.6 to 6.1. As the pH of the cross-linking solution was increased, there was a decrease in the EE of the respective MPs which may be attributed to the reduction in solubility of MZ in stronger acid medium, since MZ as a weak acid (pKa 5.8) has a negligible solubility in strong acid. MZ solubility increased by increase in pH (16). The EE of ZPGD2-MPs and CPG-MPs prepared under the same conditions were 86.1±1.7 and 83.7±1.3 respectively. The loss of MZ, for ZPG-MPs and CPG-MPs were 14% and 17% respectively, whenever determined in the cross-linking solution. Higher loss for CPG-MPs indicates exclusion of MZ which may be due to the lower degree of cross- linking and loose matrix structure.

Swellability is an indicative parameter for rapid availability of drug solution for diffusion with greater flux. It was found that SR increased by increase in polymer concentration owing to rising fluid retained capacity. Acidification of cross-linking solution was responsible for creation of a strong pectinate counter ions network (17). A low pH limited the ionization of than CPG-MPs because greater solvent penetration was taken place into the Ca-pectinate network. ZPGD2 formulation showed highest EE of 86.1±1.7%, and yield of 97.6±2.1%, smallest particle size of 526.07±6.38 µm, and lowest SR 0.88±0.37 and spherical in shape thus it was selected as optimized formulation for comparative study with CPG-MPs.

#### Polymer-drug interaction analysis using FTIR spectroscopy

**Figure 2:**
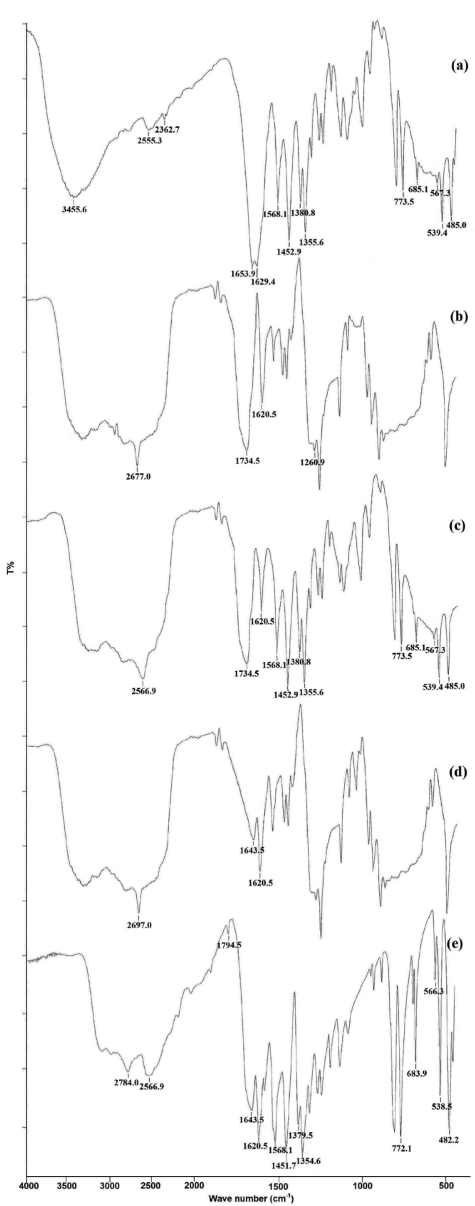
FT-IR spectra of mesalazine (a), low methoxy-pectin (b), mesalazine: low methoxy-pectin physical mixture (1:4) (c), placebo zinc pectinate gel microparticles (d), and mesalazine loaded zinc pectinate gel microparticles (e).

The characteristic IR bands for MZ at 3445 cm^−1^ were observed owing to the mutual overlapping of -NH and -OH stretching (Figure 2). The peak at 1653.9 cm^−1^ corresponds to the C=O stretching, the peak at 1629.4 cm^−1^ was assigned to NH (bend), and the peaks at 1380.8 cm^−1^ and 1355.6 cm^−1^ were attributed to -OH bending and C-N stretching respectively. The peak at 1452.9 cm^−1^ showed C=C stretching in aromatic compound. The bands in a range of 2000–3000 cm^−1^ correspond to the stretching vibrations of the hydrogen bonds in the MZ. The LM-pectin showed a broad band between 3500 and 3350 cm^−1^ due to OH stretching, a C=O vibration band of COOH group at 1734.5 cm^−1^ and the asymmetric vibration stretching of COO- at 1620.5 cm^−1^. Bands for MZ were observed clearly and almost unchanged, in the spectrum of D:P physical mixture. Spectra of placebo and drug−loaded MPs, showed a strong reduction in the LM-pectin band at 1734.5 cm^−1^, which appeard as a shoulder, and a shift to 1638 cm^−1^ of the carboxylate ion vibration stretching band, both indicative of Zn- pectinate formation (18). On the other hand, MZ characteristic peaks were slightly reduced in the spectrum of loaded MPs and appeared at 1568.1, 1451.7, 1379.5, 1354.6, 772.1, 583.9, 538.5, and 482 cm^−1^which suggest loss of drug crystallinity in the MPs and absence of high affinity interaction between LM-pectin and MZ in the MPs.

#### In vitro drug release study

The potential use of MZ loaded ZPG-MPs as CDD system was examined by performing the drug release study in SGF (pH 1.2 for 2 hrs), SIF (pH 7.4 for 3 hrs), and SCF (pH 6.8 for 7 hrs) to mimic colonic pH conditions. MPs were filled in previously optimized eudragit S 100 coated hard gelatin capsules then tested because the acrylic polymer is not soluble in acidic pH (SGF) thus it prevents drug release at pH 1.2 and starts to dissolve above pH 7. The effect of the different factors upon the release and the efficacy to target the colon were investigated.

#### Effect of the pH of cross-linking solutions and concentrations of LM-pectin

**Figure 3:**
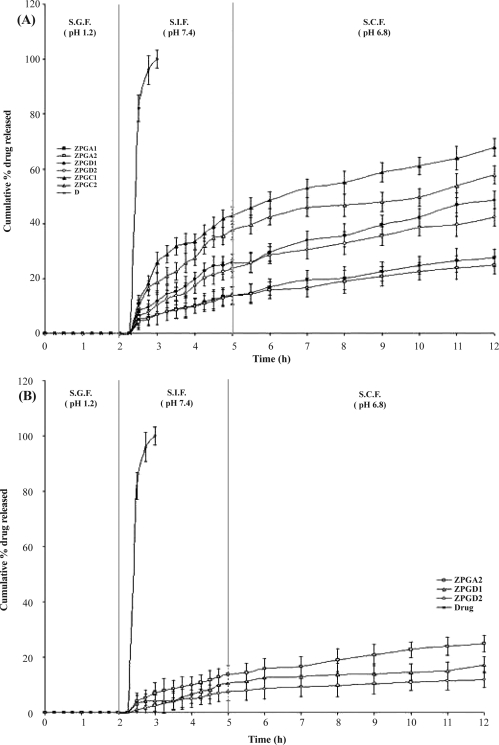
Effect of polymer concentration and cross-linking solution pH on drug release from different zinc pectinate gel microparticles in simulated GI fluids. Preparation conditions: low methoxy-pectin concentration 2% w/v+cross-linking solution pH 1.6 [▪]/3.8 [●]/6.1 [▲], and low methoxy-pectin concentration 4% w/v+cross-linking solution pH 1.6 [▫]/3.8 [○]/6.1 [△], Free drug [II] (A). Effect of cross-linking time on drug release from different zinc pectinate gel microparticles in simulated GI fluids. Preparation conditions: low methoxy-pectin concentration 4% w/v+cross-linking solution pH 1.6+cross-linking time (hrs) 6 [◊]/12 [△]/24 [▫]. Free drug [II] (B). Values are mean±s.d. (n=3).

The effect of the pH of cross-linking solutions on the drug release is shown in figure 3(A). The MPs prepared in cross-linking solution of pH 1.6 compared to the MPs prepared at pH 3.8 and 6.1 showed acceptable slow drug release. These results confirm that strong gel network of pectin was formed at lower pH below 3 (lower than LM-pectin intrinsic pKa value). Low pH probably assisted intermolecular association by reducing the charges on the polymer, thus lowering intermolecular charge- charge repulsion and also reducing the solubility of LM-pectin chain. Protonation of carboxyl groups appeared to promote conformational ordering and association by suppression of electrostatic repulsion and allowing the carbonyl groups to act as hydrogen-bond donors (19). At pH values where most of the carbonyl groups were ionized, the chain could be stiffened and extended by intermolecular electrostatic repulsion. MZ release with ZPG-MPs prepared at pH 3.8 and 6.1was faster than the one which was prepared at pH 1.6. The SR values of these MPs in SCF (pH 6.8) justify the release study. In SGF (pH 1.2) no significant amount of drug was released from MPs but 10–15% of the drug was released at pH 7.4, which may be due to the diffusion process while release in pH 6.8 could follow both diffusion and erosion mechanisms. The MZ powder filled capsules released entire drug within 2.5 hrs. Figure 3(A) also shows the effect of polymer concentration on drug release. Increasing the pectin ratio from 2% w/v to 4% w/v retarded drug release due to the increase in the density of polymer matrix and also an increase in diffusional path length that the drug molecules have transverse.

#### Effect of cross-linking time

The drug release characteristics of the formulations ZPGA2, ZPGD1, and ZPGD2 prepared at cross- linking time 6, 12, and 24 hrs respectively in same cross-linking solution of pH 1.6 and drug filled capsule are compared in figure 3 (B). Figure shows the order of the release of MZ from formulations as: ZPGD2<ZPGD1<ZPGA2. ZPGA2, ZPGD1, and ZPGD2 formulations released less than 15% up to the end of 5 hrs while drug filled capsule released entire drug within 2.5 hrs. After 5 hrs, all three formulations showed significant difference in drug release. It is clear that the drug release from the highly cross-linked formulations is slower than those of the lower cross-linked is due to the formulations. This promotion of cross-links between LM- pectin chains by the Zn (CH_3_ COO)_2_. The SR values at pH 6.8 for ZPGD2, ZPGD1, and ZPGA2 are 0.88 ± 0.32, 2.68 ± 0.28, and 1.49 ± 0.24 respectively and these values support the results for drug release.

#### Effect of the counter-ion type

**Figure 4:**
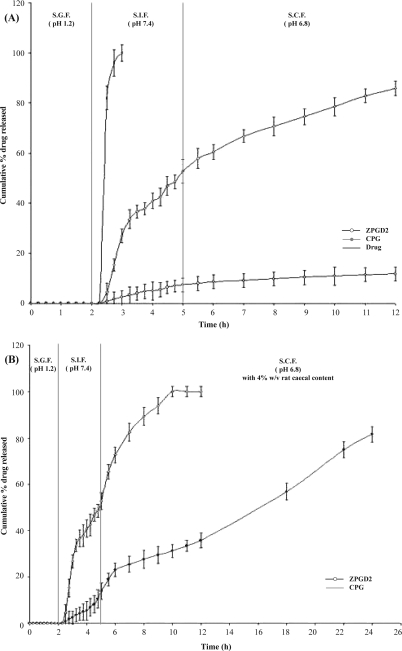
Effect of the type of cross-linking agent on drug release from zinc pectinate gel microparticles and calcium pectinate gel microparticles in simulated GI fluids. Preparation conditions: low methoxy-pectin concentration 4% w/v+cross-linking solution of pH 1.6+cross-linking time of 24 hrs+cross-linking agent Zn(CH_3_COO)_2_ [○]/CaCl_2_ [◊], Free drug [II] (A). Effect of rat caecal content on drug release from zinc pectinate gel microparticles and calcium pectinate gel microparticles in simulated colonic fluid (pH 6.8). Preparation conditions: low methoxy-pectin concentration 4% w/v+cross-linking solution pH 1.6+cross-linking time 24 hrs+cross- linking agent Zn(CH_3_COO)_2_ [○]/CaCl_2_ [◊], (B). Values are mean±s.d. (n=3).

The drug release profiles from ZPG-MPs and CPG- MPs were very different in the intestinal and colonic medium. Figure 4 (A) shows that formulation ZPGD2 released the drug in a more controlled manner as compared with formulation CPG. The release from CPG-MPs became steady after 3.25 hrs which could be due to swelling and hydration of MPs by the medium, thus showing its hydrophilic nature (12). ZPG-MPs showed slower drug release pattern in SIF (pH 7.4) characterized at the end of the equilibrium. It is possible that negligible swelling of occurred in intestinal medium. Before entrance to colon CPG-MPs showed 52.73% of drug release while ZPG-MPs showed only 7.35%. The statistical comparison of the percent of drug×from both formulations before entrance in colon showed significant differences (p<0.0001). The t_50%_, values of CPG-MPs and ZPG-MPs were 4.9 hrs and more than 12 hrs respectively. These findings suggest that the Ca^2+^ ions form loose linkages with carboxyl groups in LM-pectin chains compared to Zn^2+^ ions.

#### In vitro drug release study in the presence of RCC

To improve the relevance of the findings on the in vitro drug release study, RCC were added in dissolution media (20). The in vitro release of MZ from ZPGD2 and CPG-MPs in the presence of 4% w/v RCC in SCF showed faster drug release at different time intervals in comparison with release study in the absence of RCC (Figure 4(B)). This finding could be attributed to the various anaerobic bacteria present in caecal content responsible for digestion/degradation of LM-pectin and release of the drug from MPs. The t_50%_ value of CPG-MPs was The effects of different important variables on 3–4 folds greater than ZPG-MPs.

#### Gamma scintigraphy

**Figure 5:**
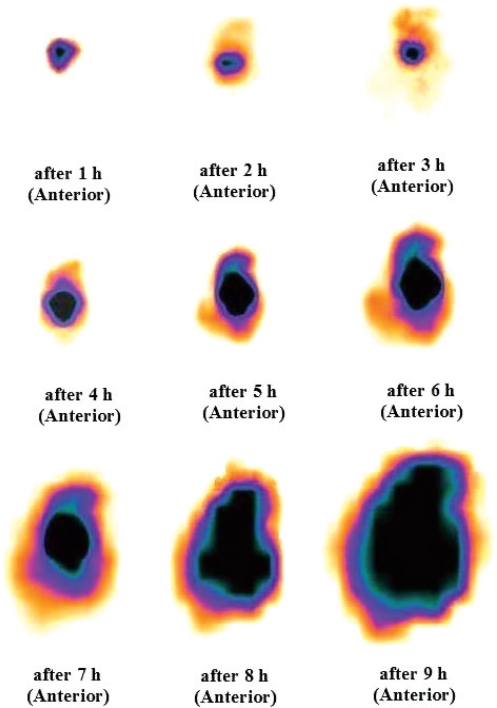
Gamma scintigraphic images of mesalazine loaded zinc pectinate gel microparticles in rabbit at different time intervals.

Gamma scintigraphy is the most useful technique to evaluate in vivo behavior of dosage forms in animals and humans since it was first employed to investigate in vivo functionality of tablets and capsules (21). The scintigraphy of the optimized formulation ZPGD2 (92±0.8% Radiolabeling efficiency) filled in enteric coated capsule was performed using rabbits (animal model) in order to establish its colon targeting potential. From the scintigraphic images (Figure 5) it can be interpreted that the capsule was completely intact in the stomach up to 2 hrs. Images indicate that when capsule containing ZPG- MPs was in the small intestine the theradioactivity was concentrated in a very small area indicating that little release had occurred. The mean transit time from stomach to colon was found to be 6.0±0.47 hrs. The capsule started to disintegrate in colon after 6.5 hrs. Once the capsule entered the ascending colon, there was considerable spreading of radioactivity from ascending colon toward the transverse colon which was most likely caused by the action of the bacterial enzymes in the colon degrading the pectin and accelerating the release of radioactivity. Scintigram shows the residence of MPs in colon more than 9 hrs. These results showed that ZPGD2 formulation may be useful for targeting MZ to the colon.

## CONCLUSION

The effects of different important variables on the characteristics of MPs prepared by ionotropic gelation technique were examined. On the basis of results, low pH of the cross-linking solution, increased cross-linking time and Zn^2+^ counter-ions in comparison with Ca^2+^ counter-ions increased the strength of the pectinate gel which reduced the swelling and drug release. The entrapment of MZ (weak acid), was significantly enhanced by the drop in pH of the cross-linking solution (maximum entrapment at pH 1.6) and also by increase in the polymer concentration. Finally, MPs were evaluated using enteric coated capsules for the drug release under conditions mimicking the overall GI tract (pH 1.2 then 7.4) and the colon (pH 6.8 with 4% w/v RCC). Gamma scintigraphy study of ZPG-MPs using enteric coated capsules, demonstrated that ZPG-MPs reached the colon with almost all the drug loading dose. It is concluded that designed formulation have the potential to serve as a colonic delivery system.
